# Status of gastric lavage in neonates born with meconium stained amniotic fluid: a randomized controlled trial

**DOI:** 10.1186/s13052-015-0194-7

**Published:** 2015-10-31

**Authors:** Lokraj Shah, Gauri Shankar Shah, Rupa Rajbhandari Singh, Hanoon Pokharel, Om Prakash Mishra

**Affiliations:** Department of Pediatrics, B.P. Koirala Institute of Health Sciences, Dharan, Nepal; Department of Obstetrics and Gynaecology, B.P. Koirala Institute of Health Sciences, Dharan, Nepal; Department of Pediatrics, Institute of Medical Sciences, Banaras Hindu University, Varanasi, India

**Keywords:** Gastric lavage, Neonates, Meconium stained amniotic fluid

## Abstract

**Background:**

Neonates born with meconium stained amniotic fluid (MSAF) can develop feed intolerance during first few days of post -natal period. A randomized controlled trial was conducted with the objectives of to find out the incidence of feed intolerance in vigorous neonates with MSAF who received gastric lavage (GL) as compared to those in whom it was not performed.

**Methods:**

This was a randomized controlled trial on 500 neonates satisfying the inclusion criteria, 230 were allocated to GL and 270 to no lavage group through computer generated random numbers.

**Results:**

No significant difference in the incidence of vomiting was found between GL and no lavage group (8.7 % vs 11.5 %, *p* = 0.305). Feed intolerance had no relationship with gestational age, gender, birth weight and mode of delivery. No neonates of GL group developed any complications related to the procedure.

**Conclusion:**

Thus, it may be concluded that gastric lavage is not required in neonates born with MSAF.

## Background

Passage of meconium is an eventual event in the post- natal period [1]. The incidence of meconium stained amniotic fluid (MSAF) ranges from 5 to12 % [2, 3]. A proportion of infants born with MSAF may swallow meconium and develop nausea, retching, vomiting, poor sucking, and secondary aspiration of meconium following vomiting in early neonatal period. Meconium in stomach acts as a chemical irritant, and may interfer with gastric function and causes undigested milk curds and feeding problems^2^. Feeding problems at the first feed have been reported to be 2.8 times more frequent in neonates born with MSAF, regardless of the consistency of meconium [4].

On the other hand, performing gastric lavage (GL) at birth for any indication is also not a very safe procedure as it may be associated with complications like apnea, bradycardia and injury to nasal cavity, oesophagus and stomach [5, 6]. Further, it can cause development of long-term visceral hypersensitivity and an increased prevalence of functional intestinal disorders in later life [7]. Previous few reports [8–10] have shown that there were no significant differences in the feeding problems in neonates in whom GL was performed in comparison to those where it was not done. However, GL is done routinely in neonates born with MSAF at most places. It is still being used at our centre and that too without any much scientific evidence of its beneficial effect.

Therefore, the present study was undertaken to know the utility of GL in vigorous late preterm and term newborns born through MSAF in comparison to those who did not receive as a primary outcome measure; and also to find out any procedure related complications such as apnoea, bradycardia and injury to organs as secondary outcome.

## Methods

### Study design

This was a non-blinded randomized controlled trial carried out at the Departments of Pediatrics and Adolescent, Medicine and Obstetrics and Gynaecology, B.P.Koirala Institute of Health Sciences, Dharan, Nepal between the period of July 2013 to June 2014. Risk of feeding problems at the first feed have been reported to be 2.8 times more frequent in neonates born with MSAF [4], so taking odds ratio of 2.8, with 80 % power of study and α error at 5 %, the sample size calculated was found to be 462 and with addition of about 10 % cases lead to a total of 500 cases.

### Subjects

All vigorous neonates born through thick MSAF either by vaginal route or lower segment caesarean section at ≥34 weeks and birth weight ≥ 1800 g were included. A vigorous neonate was defined as one with strong respiratory effort, good muscle tone and heart rate >100 per min. The neonates with respiratory distress, requiring oxygen or with major congenital malformations were excluded. The clinical trial was registered with registration number CTRI/2014/09/004988. Registration was applied before start of the study but the number was assigned before completion. The protocol of the study was approved by Institute Ethics Committee and written informed consent was obtained from parents of each neonate included in the study.

### Methods

Randomization was done by computer generated random numbers. Newborns were randomized in one of the two groups: gastric lavage and no lavage group. A nursing staff not involved in the trial did the allocation concealment by keeping the random numbers in serially opaque sealed envelopes which was open soon after delivery to enroll the case in a particular group.

One of the pediatric resident trained in neonatal resuscitation attained the deliveries and recorded the data. Gastric lavage with 10 ml/kg of normal saline was done using a 20 ml syringe and 6 G nasogastric tube in aliquots of 10 ml in neonates of GL group. Neonates in control group were not given gastric lavage. The GL was done after routine care given at place of delivery. Rest of the care at birth was same in both the groups. All babies were monitored for heart rate, respiratory rate, apnoea, bradycardia (heart rate <80/min), cyanosis and local trauma to nostrils and oral cavity due to procedure. A baseline abdominal girth at umbilicus level was recorded. All babies were advised exclusive breast feeding within 30 min of birth, as per our hospital protocol. Thereafter, neonates were shifted to postnatal wards for rooming in with the mother and monitoring for the first 48 h.

For the purpose of study, vomiting was defined as expulsion of gastric contents with effort which could be projectile also. Regurgitation was defined as effortless expulsion of milk during or immediately after feeding. Criteria for feeding intolerance was adopted as reported by Ameta et al.^8^ It included more than 2 vomiting in any 4 h period, or >3 in 24 h and/or, abdominal distension defined as increase in abdominal girth of >2 cm from base line and/or gastric residual volume >2 ml undigested milk or bilious in colour.

### Statistical analysis

The data were analyzed using SPSS version 20. The data following Gaussian distribution, Student’s t- test and of non- Gaussian distribution, Mann -Whitney *U* test were used for comparison of two groups. For comparison of categorical variable, Chi-square test was used, and Odds ratio was calculated at 95 % confidence interval, A p value of < 0.05 was considered as significant.

## Results

**Fig. 1:**
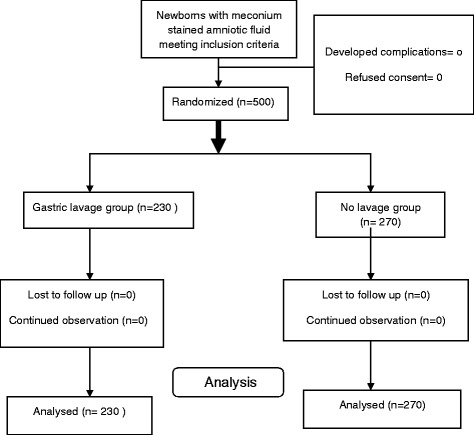
Study flow of neonates

A total of 10,797 deliveries occurred and 3312 (30.7 %) neonates were born wih MSAF during the study period; only 500 (15 %) vigorous neonates were enrolled as per sample size calculation. They were randomized as per serially arranged computer generated random numbers, and thus 230 were allocated to GL group and 270 to no lavage group. All the patients enrolled in the study were analysed (Fig. 1).

The basic characteristics and outcome measures are presented in Table 1. The mean birth weight, gestational age, gender distribution, mode of delivery, abdominal girth and median Apgar score at 5 min were comparable between the two groups. As regard to outcome, 20 (8.7 %) of GL and 31 (11.5 %) of no lavage group developed vomiting (*p* = −.0-305, Odds ratio 1.362, 95 % CI 0.754–2.462). Overall, feed intolerance was found in 51 neonates and it did not differ significantly in relation to gestational age, gender, birth weight and modes of delivery (table). No complications of nasogastric tube insertion such as apnoea, bradycardia and local tissue trauma were observed in GL group.

**Table 1:** Baseline characteristics and outcome of neonates

Variables		Gastric lavage group (*n* = 230)	No lavage group (*n* = 270)	*p*-value
Gestational age (weeks) (mean ± SD)		37.3 ± 1.5	37.6 ± 1.3	0.080*
Birth weight (g) (mean ± SD)		2748 ± 450	2835 ± 425	0.210*
Gender	Male	125 (54.3 %)	130 (48.1 %)	0.331**
	Female	105 (45.7 %)	140 (51.9 %)	0.354**
Mode of delivery	Vaginal	125 (54.3 %)	156 (57.8 %)	0.614**
	LSCS	105 (45.7 %)	114 (42.2 %)	0.494**
Apgar score median (IQR) 5 min		8(7–9)	8(7–9)	0.615***
Abdominal girth (cm) mean ± SD		25 ± 0.5	24 ± 1.0	0.910*

*n* number of cases, *LSCS* lower segment caesarean section, *IQR* inter quartile range

*Student’s *t* test, **Chi-square test, *** Mann Whitney- *U* test

## Discussion

Gastric lavage in neonates with MSAF is still a routine practice in neonatal units in order to avoid vomiting and subsequent aspiration of meconium and gastric contents. The incidence of MSAF in our study was higher than other reports [2, 3], as this is the only tertiary- care centre in eastern region of Nepal where cases with complicated deliveries are referred and often in late stage from the periphery. Mothers present with foetal distress and thus neonates were born with MSAF. We did not find any significant difference in primary outcome in the form of vomiting or any form of feeding intolerance between two groups. Narchi and Kulaylat [11] reported that 4.7 % (13 of 275) of their cases developed feeding problems in whom lavage was not done as compared to stomach wash group (227 neonates) which had no feeding problems or secondary meconium aspiration. Other studies reported incidence of feed intolerance in the range of 6.7–9.7 % in lavage as compared to 10.3–10.7 % in no lavage group, and again the differences being insignificant. It may be because of the fact that vigorous neonates have lesser duration of exposure to meconium in-utero as compared to non-vigorous. Further, early feeding in post- natal period dilutes the meconium and thus causes less irritation to gastrointestinal tract. We also observed that overall feeding intolerance also remained unchanged in relation to gestational age, gender, birth weight and mode of delivery.

No association of feeding intolerance with gender of neonates was found, which is similar to the findings of Cuello-Garcia et al. [12]. By contrast, Wiswell et al. [13] documented male neonates to be more prone to have intolerance than females. No association of birth weight and gestation with feed intolerance was in accordance with the findings of Ameta et al. [8].

No procedure related complications were seen in any case. Thus, there is consistent evidence that GL is of any benefit as regard to occurrence of feeding intolerance in vigorous neonates with MSAF. Still most neonatologists are still hesitant to change their practice of performing the stomach wash in these neonates. Although stomach wash has been mentioned as a part of routine care in babies with MSAF [14]. This is the time that attitudinal change is required regarding this procedure. It will not only reduce the financial burden on the family by reducing the cost of consumables like syringes, nasogastric tube and normal saline but also put less work load on the nursing staff. This requires support and counselling to the parents and also feeding assistance by nursing staff and physicians in neonatal units if at all any problems occur.

The limitations of this study are that there was relatively shorter period of observation (48 h) during post- natal period and it may be possible that some of the neonates might have developed feeding intolerance after this leading to under reporting of problems. Secondly, being open level study, investigator knew the assignment group thus leading to observer bias. However, we enrolled good number of cases in each treatment arm, therefore it can be concluded that gastric lavage in neonates born with MSAF should not be undertaken routinely.

## Conclusion

As such no gastric lavage is required in vigorous neonates born through MSAF to avoid feeding intolerance. This will reduce the cost of care and save the time of paramedical staff.
